# Transition Dynamics of a Dentate Gyrus-CA3 Neuronal Network during Temporal Lobe Epilepsy

**DOI:** 10.3389/fncom.2017.00061

**Published:** 2017-07-11

**Authors:** Liyuan Zhang, Denggui Fan, Qingyun Wang

**Affiliations:** ^1^Department of Dynamics and Control, Beihang University Beijing, China; ^2^Department of Information and Computing Science, School of Mathematics and Physics, University of Science and Technology Beijing Beijing, China

**Keywords:** temporal lobe epilepsy, dynamical transition, dentate gyrus-CA3 network, glutamatergic receptor, GABAergic receptor

## Abstract

In temporal lobe epilepsy (TLE), the variation of chemical receptor expression underlies the basis of neural network activity shifts, resulting in neuronal hyperexcitability and epileptiform discharges. However, dynamical mechanisms involved in the transitions of TLE are not fully understood, because of the neuronal diversity and the indeterminacy of network connection. Hence, based on Hodgkin–Huxley (HH) type neurons and Pinsky–Rinzel (PR) type neurons coupling with glutamatergic and GABAergic synaptic connections respectively, we propose a computational framework which contains dentate gyrus (DG) region and CA3 region. By regulating the concentration range of N-methyl-D-aspartate-type glutamate receptor (NMDAR), we demonstrate the pyramidal neuron can generate transitions from interictal to seizure discharges. This suggests that enhanced endogenous activity of NMDAR contributes to excitability in pyramidal neuron. Moreover, we conclude that excitatory discharges in CA3 region vary considerably on account of the excitatory currents produced by the excitatory pyramidal neuron. Interestingly, by changing the backprojection connection, we find that glutamatergic type backprojection can promote the dominant frequency of firings and further motivate excitatory counterpropagation from CA3 region to DG region. However, GABAergic type backprojection can reduce firing rate and block morbid counterpropagation, which may be factored into the terminations of TLE. In addition, neuronal diversity dominated network shows weak correlation with different backprojections. Our modeling and simulation studies provide new insights into the mechanisms of seizures generation and connectionism in local hippocampus, along with the synaptic mechanisms of this disease.

## Introduction

A great many *in vivo* and *in vitro* studies focus on revealing the mechanism underlying epilepsy, and significant progresses have been made in understanding the causes of temporal lobe epilepsy (TLE). TLE is the most common form of localization-related epilepsy in adults accounting for approximately 60~80% of all patients with epilepsy (Tatum, 2012). It is a chronic neurological disorder that is caused by various factors including neuron loss (Margerison and Corsellis, 1966), vulnerability of mossy cells, hippocampal sclerosis, and excessive expression of brain derived neurotrophic factor (BDNF; McNamara and Scharfman, 2010). One of the most important goals in TLE research today is to address the underlying mechanisms that contribute to seizures (Liotta et al., 2011; Curia et al., 2014). Another important issue is establishing the accurate network to understand the transition from normal state to morbid state. Temporal lobe is the most epileptogenic area of brain (Avoli et al., 2002). Focal seizure may be originated from internal structures such as hippocampus, which consists of dentate gyrus (DG) region, CA3 region, CA1 region, and subiculum (Lazarov and Hollands, 2016). In the field of epilepsy, the relationship between inhibition/excitation balance and epileptogenesis has been largely investigated in experimental studies (Amakhin et al., 2016). Mossy cell has excitatory glutamatergic receptors consisting of N-methyl-D-aspartate receptor (NMDAR) and α-amino-3-hydroxy-5-methyl-4-isoxazole propionic acid receptor (AMPAR; Min et al., 1998). Pyramidal neuron (Shi et al., 2001) and granule cell (Ye et al., 2005) are adjusted by AMPAR. Furthermore, inhibitory GABAergic receptor exists in fast spiking interneuron and oriens lacunosum-moleculare (O-LM) cell (Gloveli et al., 2005; Tort et al., 2007). Epilepsy has been shown to be associated with a dysfunction of inhibitory signaling mediated by GABA_A_ receptors (Sloviter, 1987). In particular, GABA_A_ receptors from epileptic tissue become less responsive to repeated activation than those from healthy tissue (Stamboulian-Platel et al., 2016). This suggests that promoting the activation of GABA_A_ receptors might be an effective antiepileptic strategy to suppress focal seizures in TLE. The well-known signaling pathways between hippocampal regions are the perforant path from entorhinal cortex (EC) to DG and the mossy fibers from DG to CA3 (Ahn et al., 2016). Excitatory signal from DG granule cell could be transmitted by mossy cell and relayed by CA3 pyramidal neuron (Amaral et al., 2007). In addition, CA3 pyramidal neuron also projects “back” to the dentate gyrus (Scharfman, 2007). Previous studies have shown a pyramidal neuron-mossy cell-interneuron-granule cell pathway is more responsible than a simply pyramidal neuron-interneuron-granule cell route (Scharfman, 1994; Kneisler and Dingledine, 1995). Some evidences show that CA3 pyramidal neuron can not only activate but also inhibit granule cell indirectly (Scharfman, 2016). However, the exact backprojection mechanism related to excitatory or inhibitory receptors is not fully revealed.

Many computational models have been proposed to describe epilepsy. These are divided to models of interictal activity and models of ictal activity (Wendling et al., 2016). Interictal epileptic spikes (IEs) are often observed in human partial epilepsies (Ratnadurai-giridharan et al., 2012). IEs correspond to transient signals presenting with a more or less sharp initial component sometimes followed by a slower, more or less pronounced, component of opposite polarity (Chauvière et al., 2012). El-Hassar et al. employed a pilocarpine model of TLE to change the glutamatergic receptors and GABAergic receptors during epileptogenesis, which has been investigated in a neural mass model (NMM) of CA1 hippocampal region (El-Hassar et al., 2007). The interictal-like activity was reproduced, which was derived from extensive simulations where model parameters were varied at the soma and dendrites of the pyramidal cell. Ratnadurai-Giridharan et al. generated template IEs using a subset of artificial paroxysmal depolarization shift (PDS) constructs (Ratnadurai-giridharan et al., 2012). NMM was also employed by Wendling et al. to simulate the transition from interictal to fast ictal activity, which further explained the impairment of dendritic inhibition (Wendling et al., 2012). For partial epilepsies, seizures are often characterized by the appearance of fast oscillation in LFPs or EEG signals (Schiff et al., 2000). High fast oscillations (HFOs) are usually associated with lower frequency range such as beta band and low gamma band (Wendling et al., 2002). Traub et al. developed a model based on postulated electrical coupling between pyramidal cell axons and reproduced very fast oscillations (VFOs; Traub and Bibbig, 2000; Scharfman and Buckmaster, 2014). Inspired by Traub's model, Munro et al. showed that an axonal plexus could also exhibit a second, distinctly different kind of VFO in a wide parameter range, and analyzed the role of electrical couplings among neurons (Munro and Börgers, 2010). Following pioneer studies about hippocampal CA3 area, scholars developed detailed models to investigate the network mechanisms underlying focal seizure initiation and propagation in other brain structures. Van et al. established a neural network with 656 neurons and found that weakly coupled cortical network could create synchronized cellular activity and seizure-like bursting (Van et al., 2005). Consequently, research on computational modeling is important for understanding the transitions of TLE, together with regional connection mechanism.

As discussed above, richness and complementarity of the various modeling approaches in the field of epilepsy research have been shown. However, in order to provide deeper understanding about discharge transitions in DG-CA3 area during TLE and investigate the backprojection mechanism behind synaptic connection, a neuronal diversity leading comprehensive network model is required. Therefore, we propose the overall structure of a comprehensive hippocampal network model that includes DG region containing granule cell, mossy cell and GABAergic interneuron, and CA3 region involving pyramidal neuron, GABAergic interneuron and O-LM cell. Cells of each region are global connection and there exists a backprojection path from CA3 to DG. In this paper, we conduct computational modeling based on biological mechanism that can reproduce the epileptiform propagation activities of pyramidal neurons, including interictal, preictal, and seizure onset, by increasing the NMDA junction conductance strength. We then compare the regional characteristics of CA3 and DG. Specifically, we change the backprojection positions and strength to study counterpropagation mechanism contributed by glutamatergic and GABAergic receptor, respectively.

## DG-CA3 network model formation

**Figure 1:**
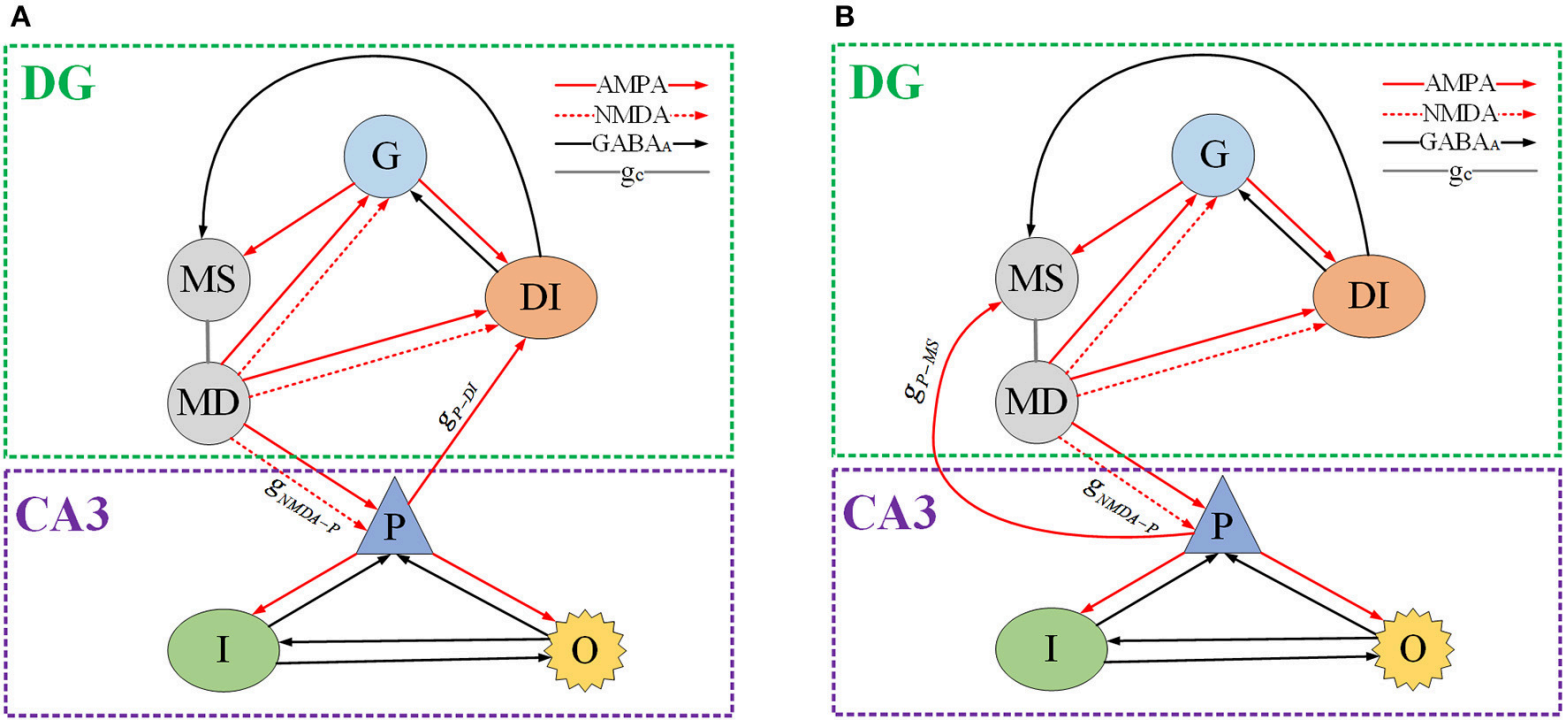
Schematic of DG-CA3 network model. G denotes granule cell. MS and MD represent mossy's soma and dendrite, respectively. DI is GABAergic interneuron of DG region. P, I, and O indicate pyramidal cell, GABAergic interneuron of CA3 and O-LM cell, severally. The red solid line with arrow means AMPA type synaptic connection, the red dash line with arrow denotes NMPA type synaptic connection. The black solid line with arrow represents inhibitory GABA_A_ coupling connection and the gray solid line g_*c*_ shows inter-compartment coupling connection of mossy cell. **(A)** The backprojection is from pyramidal cell to mossy cell's soma. **(B)** The backprojection is from pyramidal cell to GABAergic interneuron of DG.

We use Hodgkin–Huxley (HH) type models to imitate granule cell (Yuen and Durand, 1991), GABAergic interneuron, pyramidal cell, and O-LM cell (Cutsuridis et al., 2010), respectively. The mossy cell model is based on a two-compartment model which was introduced by Pinsky and Rinzel (1995). For DG region, depending on AMPA synaptic connection, granule cell innervates mossy cell and GABAergic interneuron. Then GABAergic interneuron returns inhibitory synaptic inputs to granule cell and mossy cell, through GABA_A_ synaptic connection. There exist NMDA type and AMPA type receptors in mossy cell, so as to propagate excitatory inputs to granule cell and GABAergic interneuron, respectively. Then DG region delivers excitatory synaptic inputs to CA3 region through mossy fiber path. As for CA3 region, mossy cell excites pyramidal cell relaying on NMDA and AMPA coupling connections. Pyramidal cell couples with GABAergic interneuron and O-LM cell by AMPA coupling connections, respectively. GABA_A_ synaptic connections are employed, by which GABAergic interneuron and O-LM cell could inhibit other neurons. Besides, the backprojection can locate either to soma of mossy cell or to GABAergic interneuron of DG. The DG-CA3 network schematic plot is presented in Figure 1 (See Appendix in Supplementary Material for network modeling methods).

## Results

### Effects of NMDAR from DG to CA3

**Figure 2:**
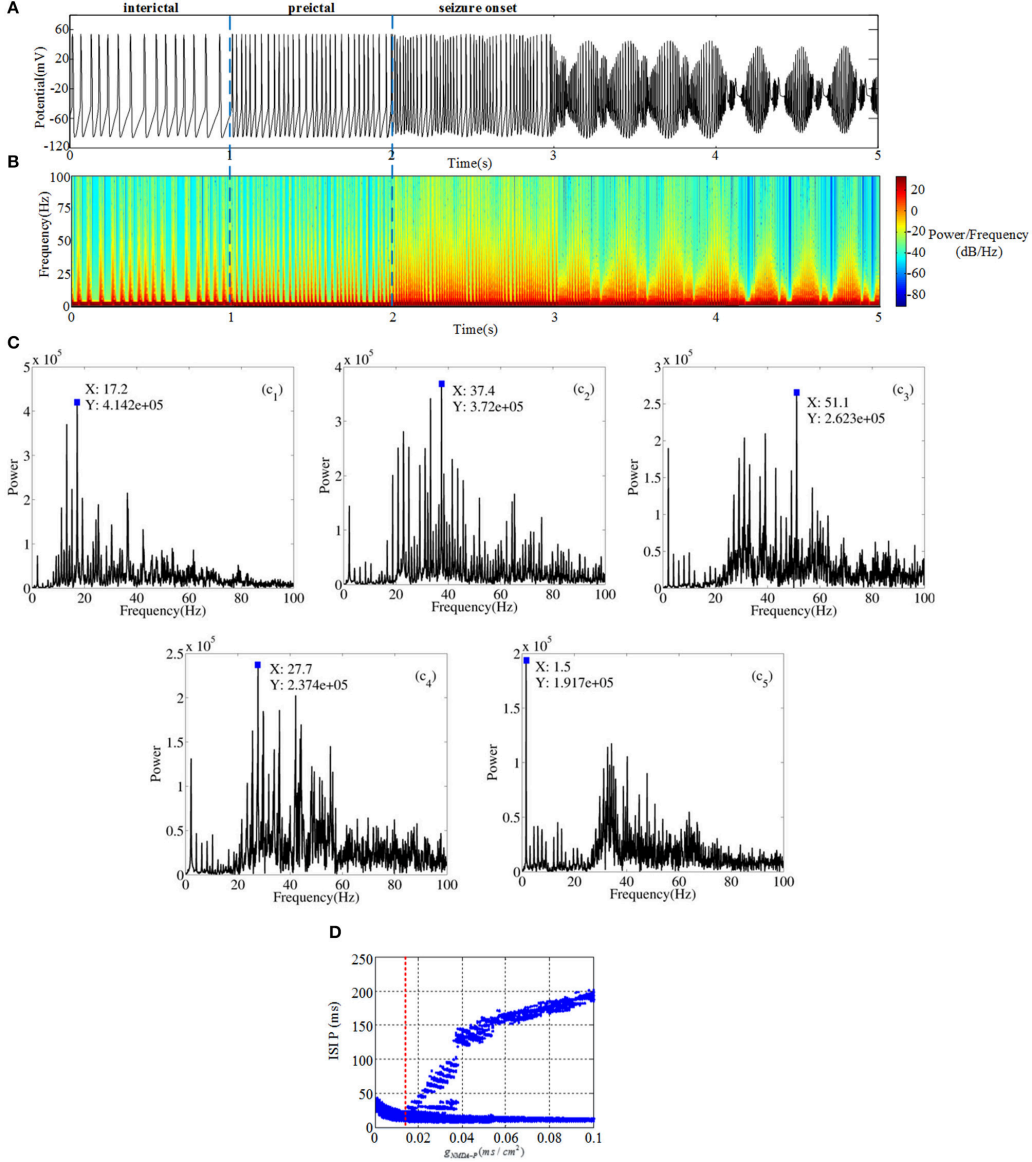
**(A)** Time series of pyramidal cell includes three different regions: interictal (0~1 s), preictal (1~2 s) and seizure onset (2~5 s). **(B)** The time-frequency graph corresponding to TLE discharge transitions. **(C)** Frequency-power plots during the transition from interictal to seizure onset. **(D)** Bifurcation diagram of pyramidal cell as the parameter g_*NMDA*−*P*_ varies.

One of the most disabling aspects of epilepsy is the unpredictable timing of the attacks. And, the study of transition from interictal to ictal processes is important for not only the insights into mechanism of epileptic seizure, but also the potential clinical applications for seizure detection and warning. Thus, we construct a micro-network to elucidate factors might be responsible for state-dependent changes in chemical receptors concentrations that subsequently lead to more seizure activities. In our study, network activity can be modified by increasing the glutamate releasing in presynaptic neuron, which leads to TLE action potential generation in the postsynaptic neuron. In this section, from pyramidal cell to mossy cell i.e., g_*P*−*MS*_, is set as 0.0001 (shown as Figure 1A). All of the neurons are set to the same external stimulus conditions. The simulation work is carried out under the circumstance of MATLAB R2013a. Simulation duration is 10 s, but we select a potential section of 1 or 2 s in order to obtain unambiguous variation. Time series, spectrograms, frequency-power figures and interspike interval (ISI) bifurcation diagram (for 10 s) are plotted to show firing behavior of pyramidal cell as the parameter changes. From Figure 2A, we can observe rich dynamical transitions as the bifurcation parameter g_*NMDA*−*P*_ increases. First, the slow interictal continuous spiking whose characteristic is 20~50 ms ISI is generated by decreasing inhibition function on pyramidal cell. As shown in Figure 2 (c1), peak valleys at 17.2 Hz own the maximum power compared with peaks. Thereafter, we conduct a moderate increase of g_*NMDA*−*P*_, pyramidal cell then displays preictal discharges presenting fast spiking with a dominant frequency about 37.4 Hz which is demonstrated in Figure 2 (c2). From Figure 2D, we observe that there exists a bifurcation point near g_*NMDA*−*P*_ = 0.002, which is the boundary of preictal and seizure onset. Following preictal discharge, seizure initiates and generates gamma (30~80 Hz) oscillations in hippocampal network. In the beginning, gamma oscillation demonstrates small bursts with dominant frequency of 51.5 Hz (shown in Figure 2 (c3)) and short ISI. Then oscillation splits into small bursts with long ISI. From Figure 2 (c4–c5) we notice that dominant frequency and amplitude show a descending trend, which implies that excessive excitatory current inhibits the neuron from discharging. In short, due to the increasing glutamatergic conductance g_*NMDA*−*P*_, pyramidal cell could present TLE discharge transitions from interictal to seizure onset.

**Figure 3:**
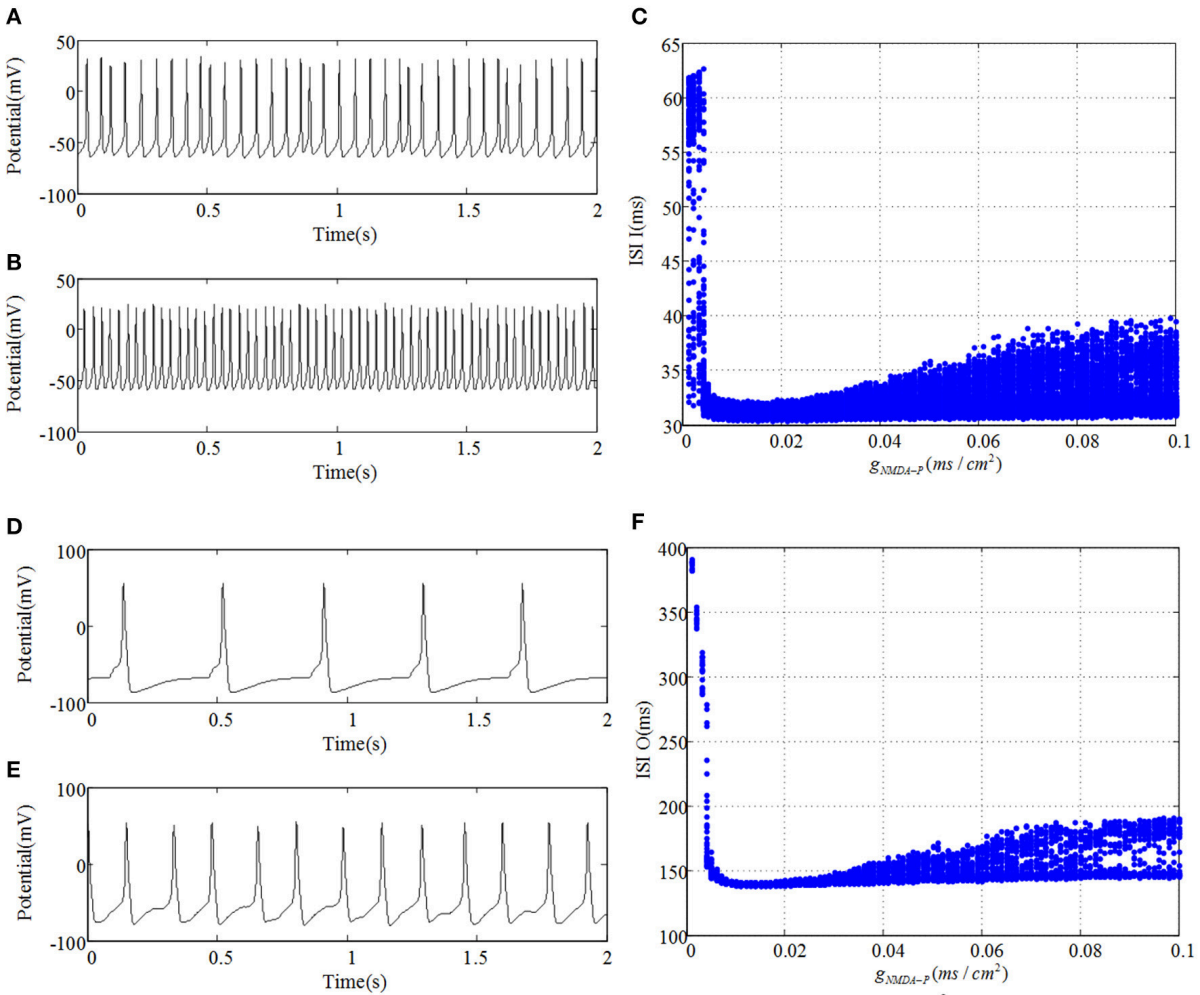
**(A,D)** plot time series of GABAergic interneuron of CA3 and O-LM cell, respectively, as g_*NMDA*−*P*_ = 0.001. Similarly, **(B,E)** severally draw curves of their membrane potentials when g_*NMDA*−*P*_ = 0.1. **(C,F)** are the corresponding ISI bifurcation diagrams.

Meanwhile, GABAergic interneuron and O-LM cell receive excitatory inputs from pyramidal cell and provide inhibitory output to CA3 region. Compared Figures 2,3, it is revealed that GABAergic interneuron and O-LM cell become excited, accompanied by an increase of glutamatergic receptor. Figures 3A,B show potential evolutions of GABAergic interneuron as g_*NMDA*−*P*_ = 0.001 and g_*NMDA*−*P*_ = 0.1, respectively. In addition, the ISI bifurcation plot is demonstrated in Figure 3C. Discharge pattern initially presents irregular spiking with longer interval, which, whereafter, transfers to fast spiking with shorter interval. Similarly, discharge transition situations of O-LM cell are displayed in Figures 3D–F. Also, potential evolution shows the process from slow spiking to fast spiking. However, the ISI of O-LM cell is obviously longer than that of GABAergic interneuron. This implies that firing rate of O-LM cell is lower than that of GABAergic interneuron, but varies dramatically in contrast. Nevertheless, two types of neurons only receive excitatory inputs from pyramidal cell so as to make the whole CA3 network become inhibitive. GABAergic interneuron and O-LM cell could not demonstrate abundant TLE discharge patterns on account of their intrinsic inhibitory mechanism. In spite of this, the enhancement of excitatory coupling strength between mossy cell and pyramidal cell could promote excitability of CA3 and induce TLE discharge transitions of pyramidal cell.

**Figure 4:**
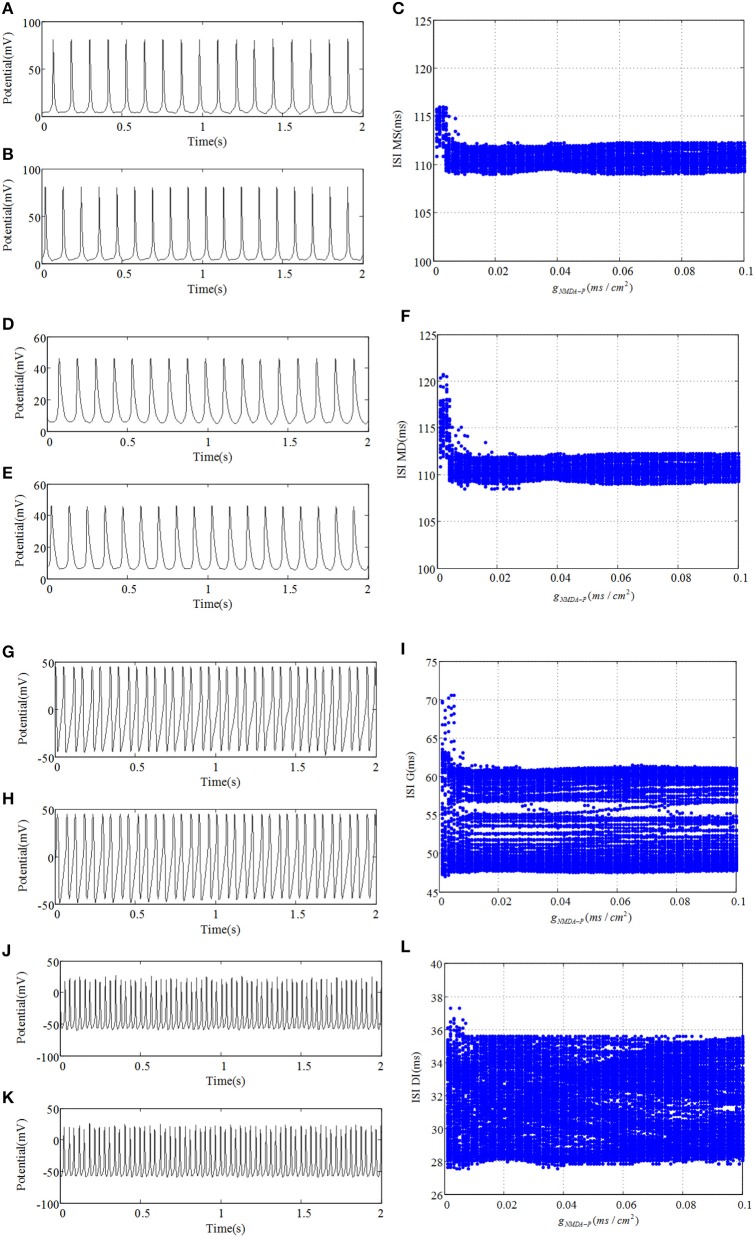
The response of DG network with weak backprojection g_*P*−*MS*_ = 0.0001 from pyramidal cell to mossy cell. **(A,D,G,J)** denote discharge potential evolutions of mossy cell's soma, mossy cell's dendrite, granule cell, and GABAergic interneuron of DG region severally when g_*NMDA*−*P*_ = 0.001. Under the condition of g_*NMDA*−*P*_ = 0.1, **(B,E,H,K)** demonstrate time series of four kinds of cells, respectively. **(C,F,I,L)** are their corresponding bifurcation diagrams.

Figure 4 demonstrates time series and ISI bifurcation diagrams of cells within DG region. As shown in Figure 4, under the condition of weakprojection from pyramidal cell to mossy cell, e.g., g_*P*−*MS*_ = 0.0001, NMDAR has little influence on discharge transitions of DG cells. Specifically, although the discharge interval of mossy cell is a little longer for the much small g_*NMDA*−*P*_ than that of relatively large g_*NMDA*−*P*_, it maintains spiking activity no matter how g_*NMDA*−*P*_ varies. Similarly, both the granule cell and GABAergic interneuron always demonstrate fast spiking activities. However, it can be observed that GABAergic interneuron oscillates with much higher frequency than that of mossy cell. This suggests that the cells within DG region possess weak correlations under the circumstance of weak backprojection which could be factored into the robustness of DG cells to the changes of NMDAR. In addition, it has been proposed that mossy cell-mediated excitation of granule cell is increased after dysfunction, causing hyperexcitability. In other aspects, changes of inhibitory control of granule cell including GABA_A_ receptors might block excitatory counterpropagation from CA3 region to DG region. Therefore, in the following section we will explore the effect of backprojection, which could contribute to the hyperexcitability or suppression of DG cells.

### Effects of backprojection from CA3 to DG

CA3 and DG are the main subfields of hippocampal formation. They receive direct innervations from EC, while previous studies believed that they were generally considered as a series of unidirectionally connected components forming the intrahippocampal “trisynaptic loop” (Avoli et al., 2002; Oliveira et al., 2011). Within the main associational network of the hippocampal formation, axon collaterals of pyramidal cell return to DG where they likely innervate mossy cells, GABAergic interneuron and granule cell (Freund, 1992; Scharfman, 2007). It is hypothesized that neuronal information emerging from the recurrent network of the CA3 region is fed back to its main driving input, the DG (Scharfman, 1994). In addition, previous physiological observation has shown that stimulation of CA3 region typically induces large inhibitory postsynaptic potentials (IPSPs) in granule cells, and it is hypothesized that this inhibition is conveyed by the excitation of GABAergic interneuron (Scharfman, 2016). However, epileptic pyramidal cell could counterpropagate morbid excitatory information to mossy cell or granule cell if GABA_A_ receptor appears inhibitory dysfunction. Thus, it suggests that CA3-DG feed-back pathway can lead to both suppression and excitation of DG region. According to this thinking, the study of effects of backprojection is carried out.

**Figure 5:**
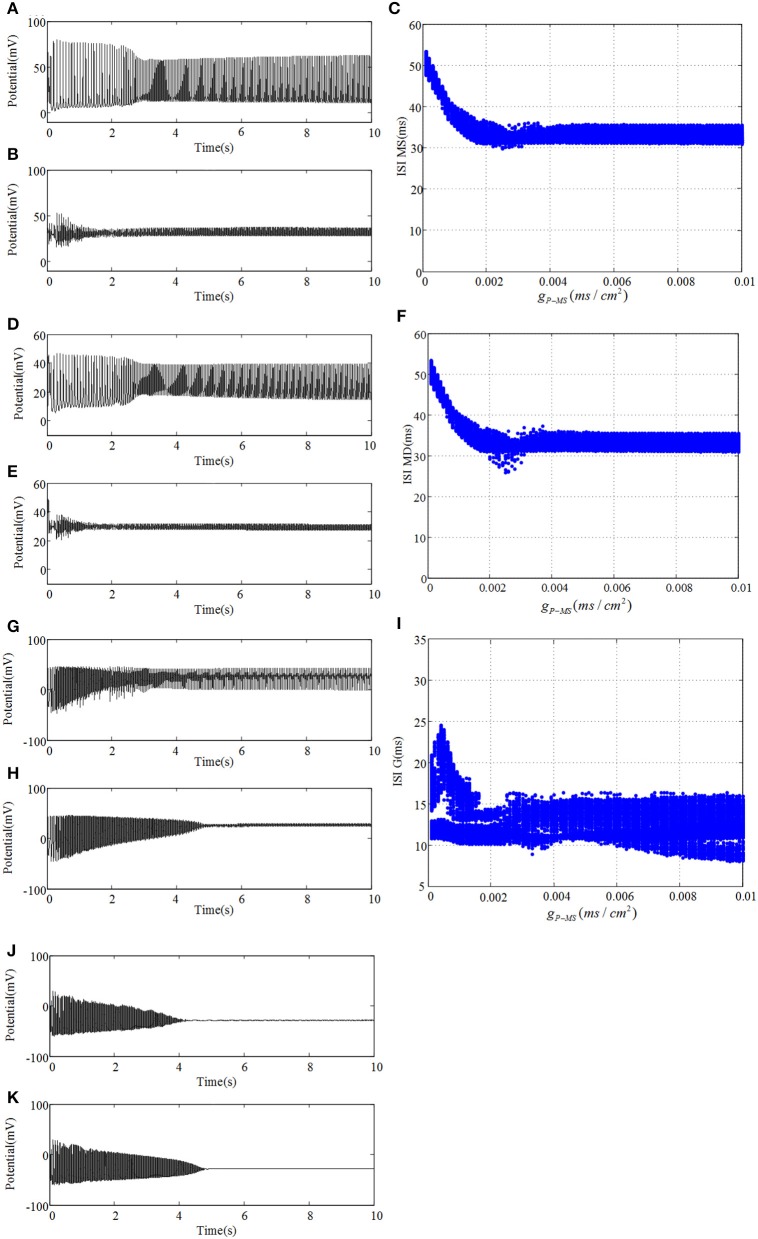
Under the condition of g_*P*−*MS*_ = 0.0001, **(A)** plots time evolution of mossy cell's soma, **(D)** denotes dendrite, **(G)** is granule cell and **(J)** presents GABAergic interneuron. **(B,E,H,K)** are their time evolution in the case of g_*P*−*MS*_ = 0.01, respectively. Besides, ISI bifurcation diagrams are demonstrated in **(C,F,I)**, respectively.

We connect the neural network according to glutamatergic type backprojection plotted in Figure 1A, it means the backprojection is from pyramidal cell to mossy cell. Figure 5 plots potential evolutions and ISI bifurcation graphs of DG with g_*P*−*MS*_ = 0.0001 and g_*P*−*MS*_ = 0.01, respectively. Besides, g_*NMDA*−*P*_ = 0.001 in this process. As time goes on, mossy cell exhibits transition from normal discharge to epileptic low-voltage fast activity, which is shown in Figures 5A–FT. What's more, as g_*P*−*MS*_ increasing, amplitude of oscillation decreases to lower values. In particular, in the beginning of Figures 5C,F we can observe distinct ISI reduction of mossy cell thereby increases with g_*P*−*MS*_ increasing until neural system reaches to the stable states. Granule cell also exhibits small-amplitude excitatory oscillation with long latency in contrast with mossy cell. GABAergic interneurons show the damped oscillations into equilibrium states. The firing latency of it can be extended when g_*P*−*MS*_ increases from 0.0001 to 0.01, which also suggests the enhancement in excitability of GABAergic interneuron.

**Figure 6:**
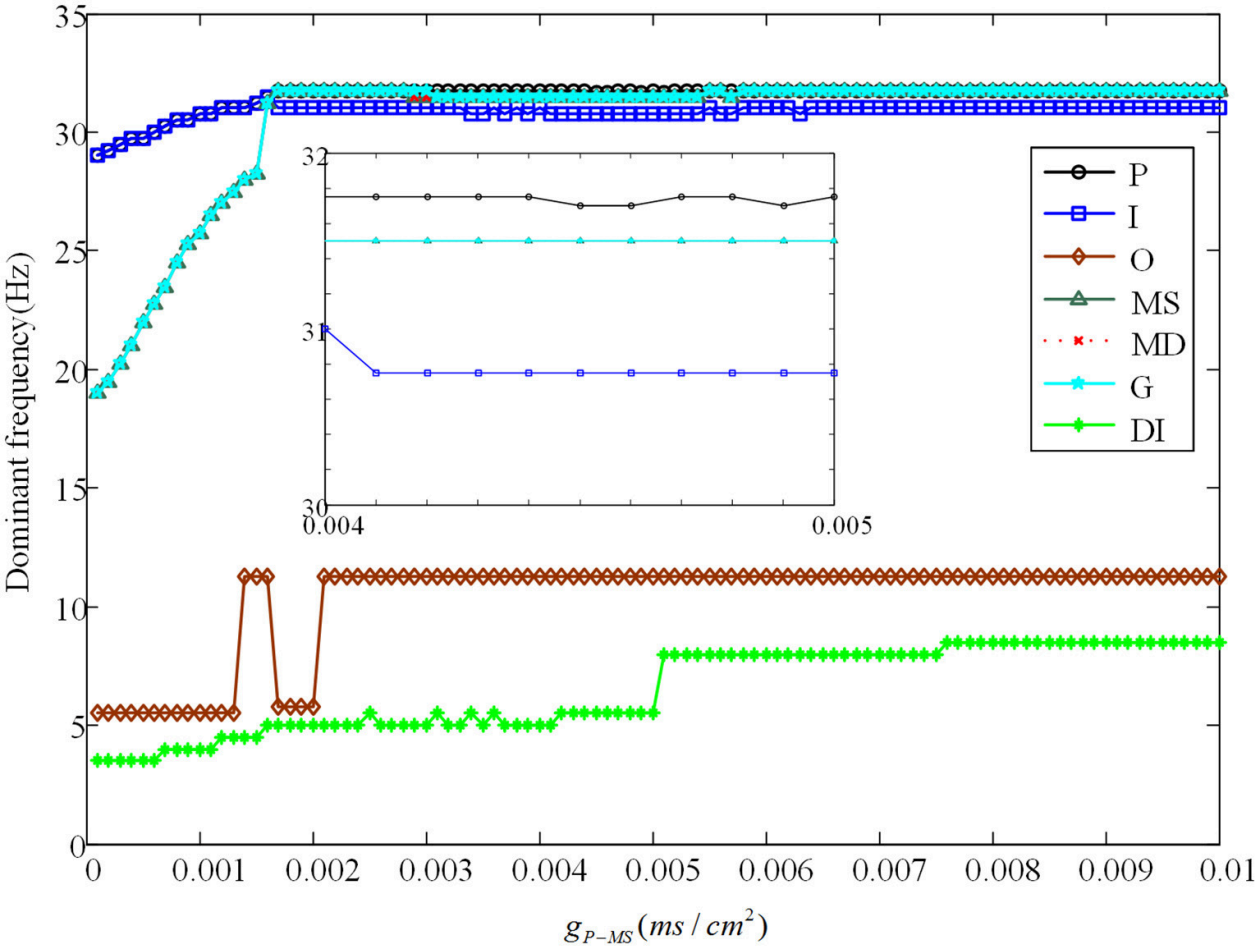
The variation of corresponding dominant frequencies. The black solid line with circles is pyramidal cell, the blue solid line with squares represents GABAergic interneuron of CA3, the brown solid line with rhombuses demonstrates O-LM cell, the green solid line with triangles is soma of mossy cell, the red dashed line with crosses illustrates dendrite of mossy cell, the cyan solid line with stars shows granule cell and the laurel-green solid line with asterisks indicates GABAergic interneuron of DG. Besides, the enlarge figure is shown with a strength interval from 0.004*ms*/*cm*^2^, to 0.005*ms*/*cm*^2^.

Besides, we analyze the corresponding dominant frequency of DG-CA3 network, which is illustrated in Figure 6. Following with the increasing of g_*P*−*MS*_, the dominant frequencies of mossy cell and granule cell vibrate at first, which then can be elevated to a higher i.e., 32 Hz, and maintained till the last. It is obvious that enhancing the backprojection coupling strength from pyramidal cell to mossy cell could promote hyperexcitability of mossy cell and granule cell, and produce low gamma oscillation. Meanwhile, GABAergic interneuron rises slowly with lower frequency value in contrast with mossy cell and granule cell. As for CA3 region, dominant frequencies of pyramidal cell and GABAergic interneuron also rise up slightly. They discharge at a relative constant frequency, such as pyramidal cell is about 33 Hz and GABAergic interneuron fires at a frequency about 32 Hz. In addition, the dominant frequency of O-LM cell increases with two jumps from 5.5 Hz to about 12 Hz. It implies that excited mossy cell could motivate CA3 region through mossy fiber even though the feed-forward coupling strength is not enhanced.

**Figure 7:**
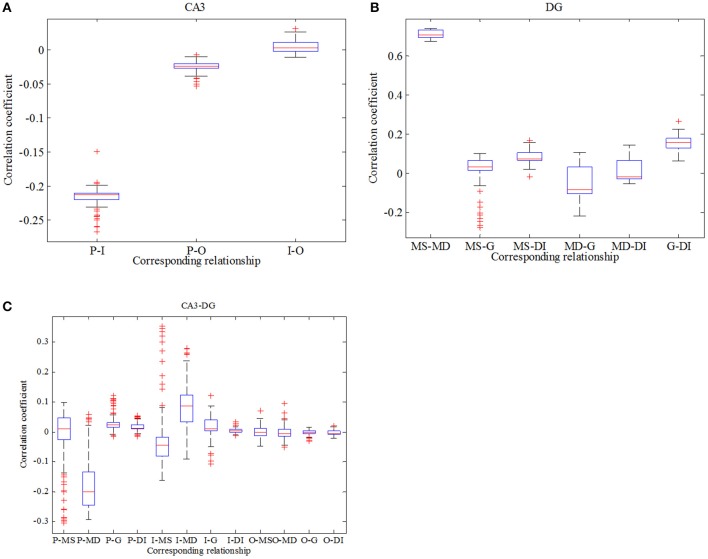
Boxplots of correlation coefficient. **(A)** reflects the relationships in CA3 region, **(B)** describes interrelations in DG region and **(C)** depicts correlation between CA3 and DG.

We also investigate the correlations among neurons. We employ correlation coefficient to estimate correlation and synchronism. Linear correlation coefficient is defined as:

(1) $$C_{xy}\left(l\right)={\langle \left(\frac{x_{i}-\bar{x}}{\sigma_{x}}\right)\left(\frac{y_{i+l}-\bar{y}}{\sigma_{y}}\right)\rangle }_{i}$$

where $\bar{x}$ and $\bar{y}$ are mean values, respectively. σ_*x*_ and σ_*y*_ represent variances separately. *l* denotes time delay (here *l* equals to zero) and 〈·〉 expresses average value. Figure 7A reflects statistical overview of correlation coefficient in CA3 region, Figure 7B describes the interrelation in DG region and Figure 7C represents the relationships between CA3 region and DG region. From Figure 7A we can clearly observe that pyramidal cell and GABAergic interneuron show stronger negative relation than that between pyramidal cell and O-LM cell. Whereas the relationship between GABAergic interneuron and O-LM is weakly positive. In the case of DG region, mossy cells are highly positively related, the correlation coefficient of which is larger than 0.6. What's more, mossy cell's soma and other neurons demonstrate weak positive correlation, while mossy cell's dendrite and the rest cells show weak negative correlations. In addition, granule cell and GABAergic interneuron coupled with each other also show a weak correlation. In the Figure 7C, we consider the relationships between regions of CA3 and DG. Firstly, pyramidal cell has positive correlations with three types of neurons within DG except for dendrite of mossy cell. Then, GABAergic interneuron of CA3 weakly and positively interacts with DG region apart from soma of mossy cell. Besides, O-LM cell has weak correlation with DG region. View the situation as a whole, pyramidal cell shows the maximum fluctuation and susceptibility, while O-LM cell is in a completely opposite situation. Mossy cell has the highest value of correlation coefficient. Good correlation between pyramidal cell and GABAergic interneuron of CA3 could be found. In addition, GABAergic interneuron of DG couples with granule cell with a relative higher correlation coefficient. Judging from Figures 5–7, we summarize that enhancing backprojection strength from pyramidal cell to mossy cell's soma could strengthen the degree of epilepsy morbidity in DG region, even though neurons do not fire hyper-synchronously, which is induced by neuronal diversity.

**Figure 8:**
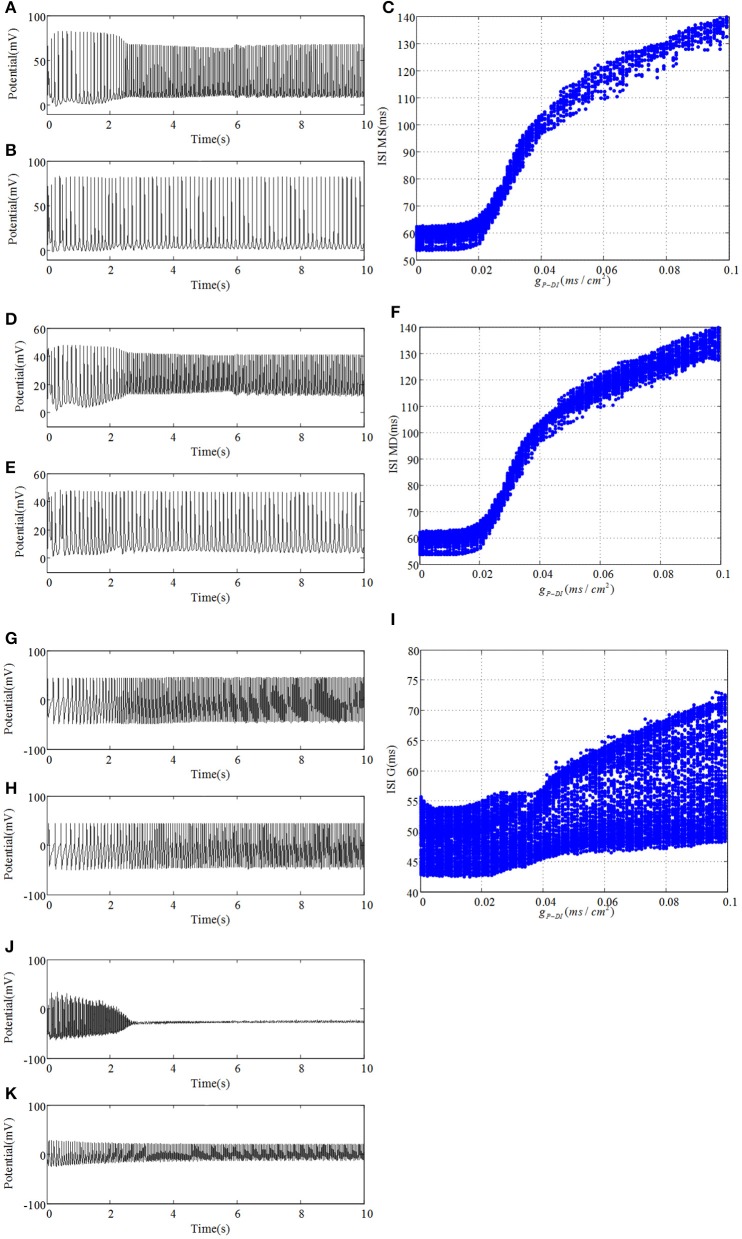
Under the condition of *g*_*P*−*DI*_ = 0.001, **(A)** plots time evolution of mossy cell's soma, **(D)** denotes dendrite, **(G)** is granule cell and **(J)** presents GABAergic interneuron. **(B,E,H,K)** are their time series in the case of *g*_*P*−*DI*_ = 0.1, respectively. Besides, ISI bifurcation diagrams are demonstrated in **(C,F,I)**.

Next, we reassemble the network in accordance with GABAergic type backprojection shown in Figure 1B, where backprojection is from pyramidal cell to GABAergic interneuron of DG region, i.e., g_*P*−*DI*_. Besides, g_*NMDA*−*P*_ = 0.001 in this process. Figure 8 presents time series and ISI birfucation graphs of DG region with the increasing of g_*P*−*DI*_. As depicted in Figures 8A,B,D,E, firing interval of mossy cell is prolonged, which means inhibitory current generated by GABAergic interneuron blocks excitatory propagation from pyramidal cell to mossy cell. Figures 8C,F give ISI evolutions about mossy cell. It is shown that mossy cell discharges with small ISI at first, then the ISI of mossy cells can be gradually enlarged when g_*P*−*DI*_ increased beyond the critical point g_*P*−*DI*_ = 0.02. As for granule cell, similar ISI variation can be observed in Figure 8I, but the oscillations seems to be more stochastic compared to mossy cells. As backprojection strength is enhanced, GABAergic interneuron of DG region leads a transition from damped oscillations to fast spiking with low amplitude, shown in Figures 8J,K, which suggests that the activity of GABAergic interneuron has been enhanced.

**Figure 9:**
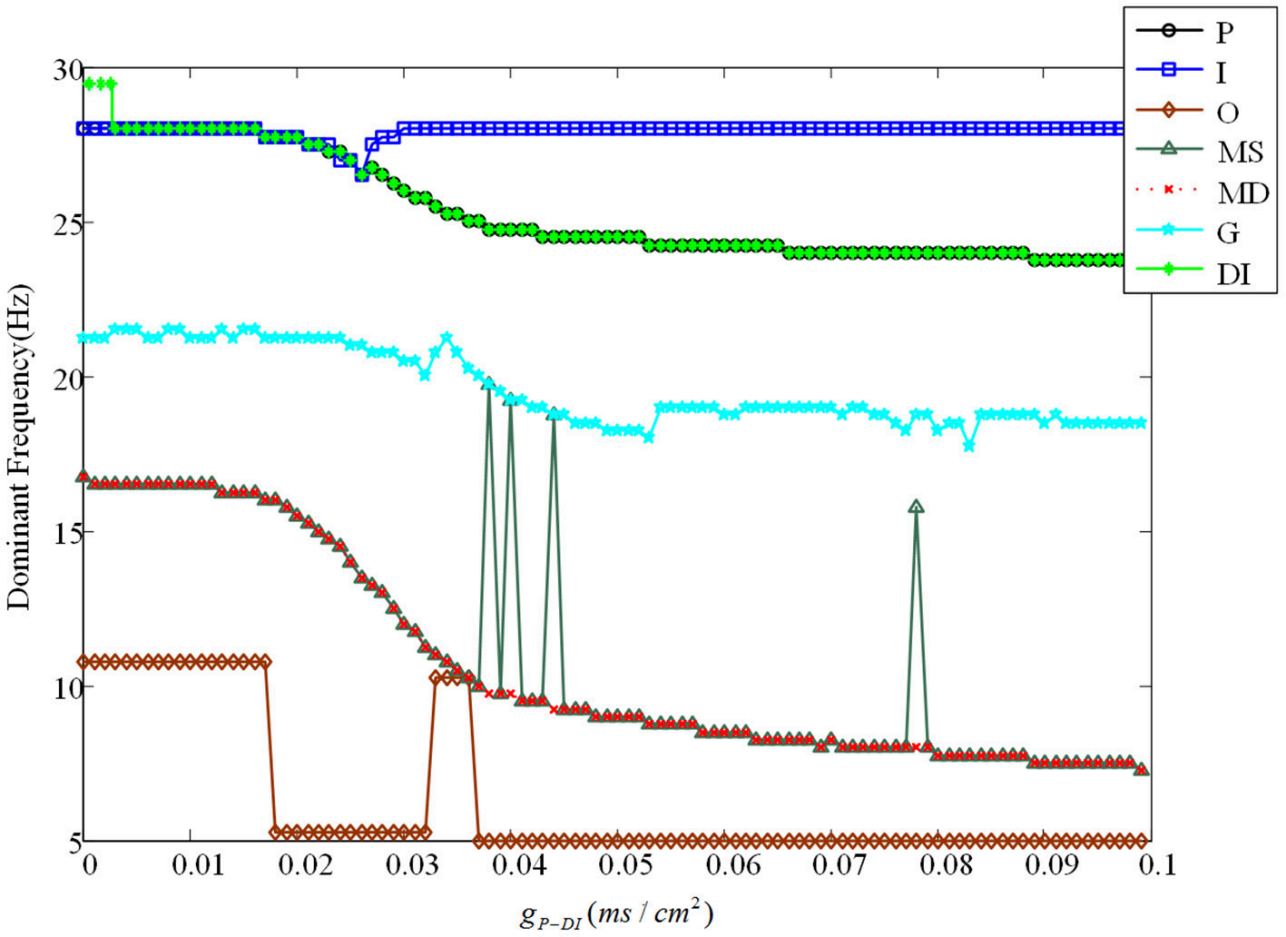
The variation of corresponding dominant frequencies. Pyramidal cell is plotted by black solid line with circles. The blue solid line with squares indicates GABAergic interneuron of CA3. O-LM cell is shown with brown solid line with rhombuses. The green solid line with triangles is soma of mossy cell and the red dashed line with crosses illustrates dendrite. In addition, the cyan solid line with stars shows granule cell and the laurel-green solid line with asterisks indicates GABAergic interneuron of DG.

Evolution of dominant frequencies is illustrated in Figure 9. Affected by GABAergic interneuron, dominant frequency of mossy cell decreases distinctly, and so does pyramidal cell. Granule cell fluctuates mildly from 21.25 to 18.5 Hz. Dominant frequency of GABAergic interneuron in CA3 basically maintains a constant as a whole, except for the outliers within *g*_*P*−*DI*_ ∈ (0.017, 0.03). Similarly, dominant frequency of O-LM cell decays from 10 to 5 Hz after several abrupt jumps. It is noteworthy that GABAergic interneuron of DG has almost the same variation trend with pyramidal cell. Although dominant frequency of GABAergic interneuron of DG declines, it discharges at a frequency of 29 Hz in the beginning and owns higher frequency, by making a comparison between Figures 6, 9, which implies that the excitability of GABAergic interneuron of DG is enhanced.

**Figure 10:**
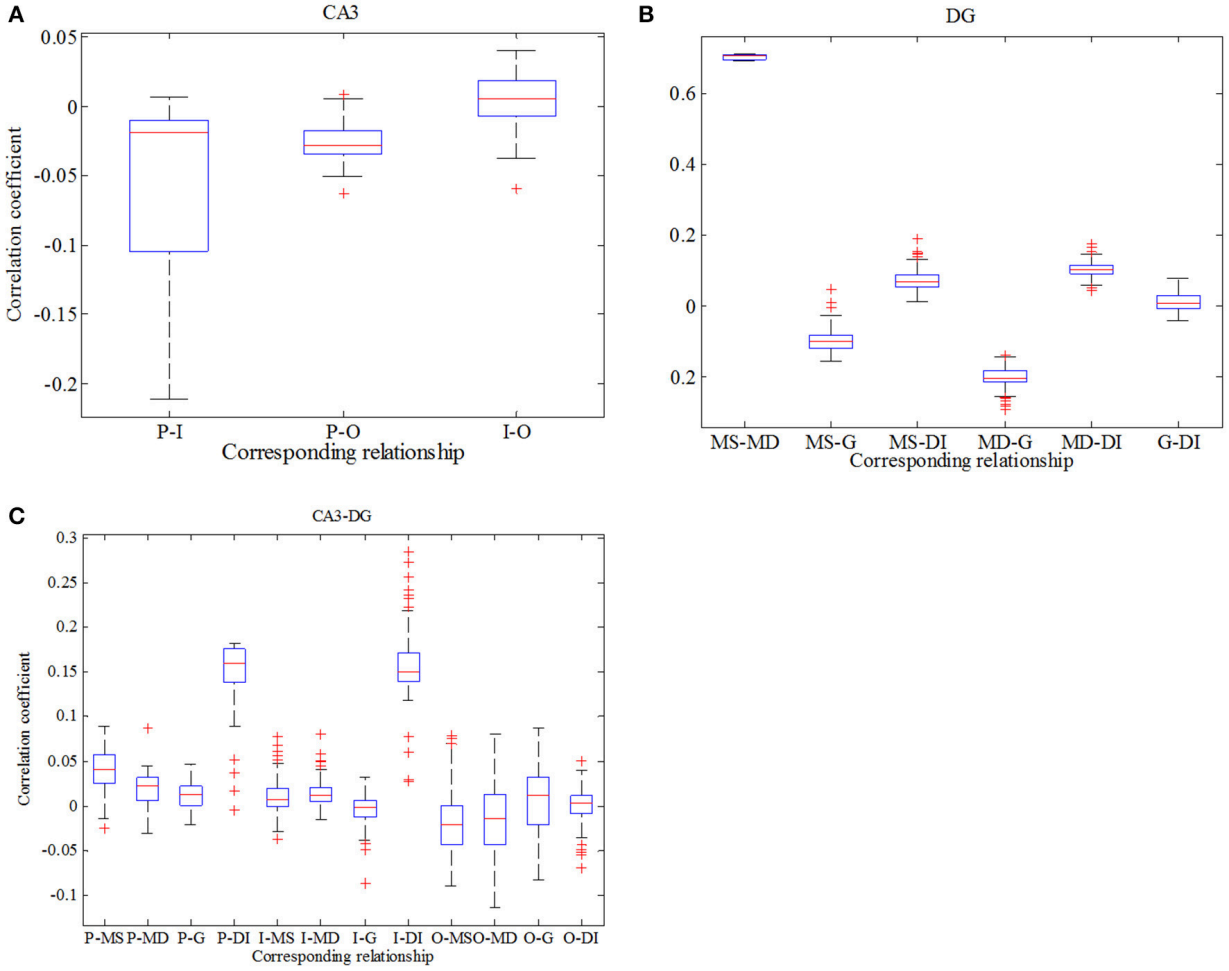
Boxplots of correlation coefficient. **(A)** reflects the relationships in CA3 region, **(B)** describes interrelations in DG region and **(C)** depicts correlation between CA3 and DG.

In order to be consistent with previous study, analysis about correlation in this case is exhibited in Figure 10. Firstly, we conclude from Figure 10A that correlation between pyramidal cell and GABAergic interneuron is negative which is weaker in comparison with Figure 7A. Similarly, pyramidal cell also weakly related to O-LM cell. Besides, GABAergic interneuron has little effects on O-LM cell. In consideration of DG region, mossy cell's soma and dendrite still have the maximum positive correlation, while the whole mossy cell has negative correlation with granule cell and positive correlation with GABAergic interneuron, respectively. Nevertheless, granule cell and GABAergic interneuron present weak correlation. At last, we study correlation between CA3 and DG. Pyramidal cell demonstrates positive correlation with DG region, which means pyramidal cell could motivate neurons of DG. In addition, we find that O-LM cell weakly correlates with DG region.

After the comprehensive comparisons between Figures 5, 10, it is proved that if pyramidal cell irritates soma of mossy cell, the latter could display hyper-excitatory and release excitatory NMDAR and AMPAR to postsynaptic neuron. Together with mossy cell, granule cell, and GABAergic interneuron of DG also demonstrate an increase in excitability, which behave as low amplitude oscillation. At the same time, CA3 region could also be slightly activated through the mossy fiber. What's more, glutamatergic type backprojection could promote dominant frequency of DG-CA3 network and then strengthen its excitability. Besides, granule cell couples with GABAergic interneuron of DG in a relative high positive correlation, when compared with pyramidal cell which synchronize negatively with GABAergic interneurons. When pyramidal cell connects DG region with GABAergic type backprojection, except GABAergic interneuron of DG, morbid excitability of DG-CA3 network decreases because of the excessive inhibitory currents. In addition, GABAergic type backprojection could decrease dominant frequency of the whole network. Thus, the activation GABAergic interneuron of DG could block counterpropagation of pathological discharges from CA3 to DG, which may prevent propagation of hyper-synchronous discharge from another perspective. At last, we can conclude that dendrite of mossy cell discharges with a negative synchronous coefficient and pyramidal cell couples with GABAergic interneuron of DG in positive correlation. Phenomenon of correlation is not obvious due to the neuronal diversity.

## Conclusion

Using a combination of TLE theory and numerical advanced models, we constructed a DG-CA3 network to explore the mechanism underlying TLE transitions and the network synaptic connection. This network model offers novel opportunities to model and study epilepsy behavior from dynamics perspective. Action potentials of different cells within the DG-CA3 network are used to describe the network activity state. Firing characteristics of action potentials are mainly reflected by the spectrograms and interspike interval (ISI). Firstly, by enhancing the coupling strength of DG-CA3 pathway, we have demonstrated complex TLE transitions including interictal, preictal, and seizure onset in pyramidal cell, meanwhile, excitability of CA3 region increases. In particular, in Figure 2 we employed the time-frequency graph to more evidently observe the changes of activity frequencies during TLE transition process. In addition, taking advantage of the connectionism of hippocampal, we have studied the effects of backprojetion during TLE transitions. On the one hand, the detailed dynamics and qualitative analysis have shown that the glutamatergic type backprojetion from pyramidal cell to mossy cell contributes to excitatory morbid discharges of DG region. On the other hand, simulation results also have indicated that the GABAergic type backprojection from pyramidal cell to GABAergic interneuron could block excitatory counterpropagation. Whereas the network acts less hypersynchronous, which is due to the neuronal diversity playing the dominant role in synchronization. Our approach may have validity only within a small range of physiological structure, nevertheless, they can give insight about experimental paradigms via simulations and analyses bridging the gap neuronal excitability, network and TLE evolution across large temporal scales. Hence, we hope that our work will be useful for further epilepsy disease analysis and be helpful for other nerve system disease.

## Author contributions

LZ, DF, and QW designed and performed the research as well as wrote the paper.

### Conflict of interest statement

The authors declare that the research was conducted in the absence of any commercial or financial relationships that could be construed as a potential conflict of interest.
