# Supplementary material for: Transition Dynamics of a Dentate Gyrus-CA3 Neuronal Network during Temporal Lobe Epilepsy
Source: Front Comput Neurosci. 2017 Jul 11;11:61. doi: 10.3389/fncom.2017.00061 (PMC5504536; doi:10.3389/fncom.2017.00061)
Supplement: Supplementary file 1 [file DataSheet1.docx]

# Appendix

Pyramidal cell: The pyramidal cell is described by the following equations (Olufsen et al., 2003):

$C\frac{dV_{P}}{dt}=g_{Na}m_{\infty}\left( V_{P} \right)^{2}h\left( V_{Na}-V_{P} \right)+g_{K}n^{4}\left( V_{K}-V_{P} \right)+g_{L}\left( V_{L}-V_{P} \right)+I_{P}$ (A1)

$\frac{dh}{dt}=\frac{h_{\infty}\left( V_{P} \right)-h}{\tau_{h}(V_{P})}$ (A2)

$\frac{dn}{dt}=\frac{n_{\infty}\left( V_{P} \right)-n}{\tau_{h}(V_{P})}$ (A3)

$x_{\infty}\left( V_{P} \right)=\frac{\alpha_{x}(V_{P})}{\alpha_{x}\left( V_{P} \right)+\beta_{x}(V_{P})}$ $\mathrm{for}x=m,h or n$ (A4)

$\tau_{x}\left( V_{P} \right)=\frac{1}{\alpha_{x}\left( V_{P} \right)+\beta_{x}(V_{P})}$ $\mathrm{for}x=h or n$ (A5)

where $C$, $V_{P}$, $t$,$g$ and $I_{P}$ denote capacitance density, membrane potential, time, conductance density and current density, respectively. The units of above variables are $\mu F/cm^{2}$, $\mathrm{mV}$, $\mathrm{ms}$, $ms/cm^{2}$ and$\mu A/cm^{2}$. Units will be omitted for brevity. The parameter values of the model are$C=1$, $V_{Na}=50$,$V_{K}=100$,$V_{L}=-67$, $g_{Na}=100$, $g_{k}=80$ and $g_{L}=0.1$.

CA3 and DG GABAergic interneurons: We employ Wang’s model for GABAergic interneurons (Wang and Buzs´aki G, 1996). Differential equations are shown as:

$C\frac{dV_{I,DI}}{dt}=g_{Na}m_{\infty}\left( V_{I,DI} \right)^{2}h\left( V_{Na}-V_{I,DI} \right)+g_{K}n^{4}\left( V_{K}-V_{I,DI} \right)+g_{L}\left( V_{L}-V_{I,DI} \right)+I_{I,DI}$ (A6)

$\frac{dh}{dt}=\frac{h_{\infty}\left( V_{I,DI} \right)-h}{\tau_{h}(V_{I/DI})}$ (A7)

$\frac{dn}{dt}=\frac{n_{\infty}\left( V_{I,DI} \right)-n}{\tau_{h}(V_{I,DI})}$ (A8)

$x_{\infty}\left( V_{I,DI} \right)=\frac{\alpha_{x}(V_{I,DI})}{\alpha_{x}\left( V_{I,DI} \right)+\beta_{x}(V_{I,DI})}$ $\mathrm{for}x=m,h or n$ (A9)

$\tau_{x}\left( V_{I,DI} \right)=\frac{1}{\alpha_{x}\left( V_{I,DI} \right)+\beta_{x}(V_{I,DI})}$ $\mathrm{for}x=h or n$ (A10)

where $C=1$,$V_{Na}=55$,$V_{K}=-90$,$V_{L}=-65$, $g_{Na}=35$, $g_{k}=9$ and $g_{L}=0.1$.

O-LM cell: For O-LM cell, we use the model described by Tort et al (Tort et al., 2008). The current-balance differential equation and dynamics equation of gating variables are given by:

$$C\frac{dV_{O}}{dt}=g_{Na}m^{3}h\left( V_{Na}-V_{O} \right)+g_{K}n^{4}\left( V_{K}-V_{O} \right)+g_{A}ab\left( V_{A}-V_{O} \right)$$

$+ g_{h}r\left( V_{h}-V_{O} \right)+{g_{L}\left( V_{L}-V_{O} \right)+I}_{O}$ (A12)

$\frac{dx}{dt}=\frac{x_{\infty}\left( V_{O} \right)-x}{\tau_{x}(V_{O})}$ $\mathrm{for}x=m,h,n,a,b or r$ (A13)

where $C=1$.3,$V_{K}=-100$, $V_{A}=-90$, $V_{h}=-32.9$,$V_{L}=-70$, $g_{Na}=30$, $g_{k}=23$, $g_{L}=0.05$, $g_{A}=16$, $g_{h}=12$ and $V_{Na}=90$.

Granule cell: We adopt a reconstruction somatic model as granule cell (Yuen and Durand, 1991). The dynamical equations of granule cell are given by:

$C\frac{dV_{G}}{dt}=g_{Na}m^{3}h\left( V_{Na}-V_{G} \right)+g_{K}n^{4}\left( V_{K}-V_{G} \right)+g_{Ca}s^{2}w\left( V_{Ca}-V_{G} \right)+g_{sk}q^{2}\left( V_{K}-V_{G} \right)+g_{L}\left( V_{L}-V_{G} \right)+I_{G}$ (A14)

$\frac{d{[{Ca}^{2+}]}_{in}}{dt}=-\frac{\left[ {Ca}^{2+} \right]_{in}}{\tau_{d}}-\frac{I_{{Ca}^{2+}}}{wzF}$ (A15)

$\frac{dx}{dt}=\alpha_{x}\left( 1-x \right)-\beta_{x}x$ $\mathrm{for}x=m,h,n,s,w or q$ (A16)

where $C=$3.4, $g_{Na}=250$, $g_{k}=40$, $g_{L}=0.025$, $g_{Ca}=1$, $g_{sk}=4.7$, $V_{Na}=45$,$V_{K}=-85$, $V_{Ca}=70$, $V_{h}=-32.9$ and$V_{L}=-67.46$.$w=0.2\mu m$ is the depth of the shell, $z$ is the valence of calcium icon, and $F$ is the Faraday constant. The calcium removal rate $\tau_{d}$ is $9ms$. The initial internal calcium concentration ${[{Ca}^{2+}]}_{in}$ and steady-state calcium concentration ${[{Ca}^{2+}]}_{in,steady}$ are all $0.1\mu m$.

Mossy cell: The mossy cell model is introduced by Pinsky and Rinzel (Pinsky and Rinzel, 1995). The dynamics equations for two compartments are given below:

$C\frac{dV_{MS}}{dt}=g_{Na}m_{\infty}^{2}h\left( V_{Na}-V_{MS} \right)+g_{K}n\left( V_{K}-V_{MS} \right)+\frac{g_{c}}{p}\left( V_{MD}-V_{MS} \right)+g_{L}\left( V_{L}-V_{MS} \right)+\frac{I_{MS}}{p}$ (A17)

$$C\frac{dV_{MD}}{dt}=g_{Ca}s^{2}\left( V_{Ca}-V_{MD} \right)+g_{KAPH}q\left( V_{K}-V_{MD} \right)+g_{KC}\chi\left( Ca \right)\left( V_{K}-V_{MD} \right)+\frac{g_{c}}{1-p}\left( V_{MS}-V_{MD} \right)$$

$+g_{L}\left( V_{L}-V_{MD} \right)+\frac{I_{MD}}{1-p}+\frac{I_{NMDA}+I_{AMPA}}{p-1}$ (A18)

$\frac{dCa}{dt}=0.13g_{KC}g_{KC}\chi\left( Ca \right)\left( V_{K}-V_{MD} \right)-0.0075Ca$ (A19)

$\frac{dy}{dt}=\frac{y\left( V_{MS,MD} \right)-y}{\tau_{y}(V_{MS,MD})}$ $\mathrm{for}y=h,n,s,c or q$ (A20)

$y_{\infty}(V_{MS,MD})=\frac{\alpha_{y}(V_{MS,MD})}{\alpha_{y}\left( V_{MS,MD} \right)+\beta_{y}(V_{MS,MD})}$ (A21)

$\tau_{y}\left( V_{MS,MD} \right)=\frac{1}{\alpha_{y}\left( V_{MS,MD} \right)+\beta_{y}(V_{MS,MD})}$ (A22)

$I_{NMDA}=\frac{g_{NMDA}S_{i}\left( t \right)}{(1+0.28\exp\left( -0.062\left( V_{MD}-60 \right) \right)\times(V_{MD}-V_{syn})}$ (A23)

$I_{AMPA}=g_{AMPA}W_{i}(t)(V_{MD}-V_{syn})$ (A24)

$\frac{dS_{i}}{dt}=\sum_{j} H\left( V_{MS,j}-10 \right)-\frac{S_{i}}{150}$ (A25)

$\frac{dW_{i}}{dt}=\sum_{j} H\left( V_{MS,j}-10 \right)-\frac{W_{i}}{2}$ (A26)

$H\left( x \right)=\left\{ \begin{aligned} 1 x\geq0 \\ 0 x<0 \end{aligned} \right.$ (A27)

where $C=$3, $g_{Na}=250$, $g_{KDR}=15$, $g_{L}=0.1$, $g_{Ca}=10$, $g_{KAPH}=0.8$ and $g_{KC}=15$.The reversal potentials are $V_{Na}=120$,$V_{K}=-85$, $V_{Ca}=140$, $V_{syn}=60$ and$V_{L}=0$. Besides, $\chi\left( \mathrm{Ca} \right)=min(Ca/250,1)$.

To ensure equity, all of the neurons receive the same initiative stimulus: $I_{P}=I_{I}=I_{O}=I_{MS}{=I}_{MD}=I_{G}=I_{DI}=5\mu A/cm^{2}$. In addition, the unmentioned coupling conductance is set to $0.001ms/cm^{2}$.

Synaptic model: We adopt the synaptic model proposed by Ermentrout and Kopell (Ermentrout and Kopell, 1998). The synaptic gating variable $s$ is defined as $0\leq s\leq1$ and obeys rule as below:

$\frac{ds}{dt}=\rho\left( V \right)\frac{1-s}{\tau_{R}}-\frac{s}{\tau_{D}}$ (A28)

where $\rho$ represents Heaviside function:

$\rho\left( V \right)=\frac{1+tanh(V/4)}{2}$ (A29)

and $\tau_{R}$ and $\tau_{D}$ are the rise and decay time constants, respectively. Then, the synaptic current is given by:

$g_{ij}s_{i}(t)(V_{rev}-V_{j})$ (A30)

where $g_{ij}$denotes the conductance, $s_{i}$ denotes the gating variable and $V_{rev}$ is synaptic reversal potential. In addition, $V_{j}$ is the membrane potential of neuron $j$. For AMPAR-type synapses, we set $\tau_{R}=0.1$, $\tau_{D}=3$ and $V_{rev}=0$; for GABA_A_R-type synapses, $\tau_{R}=0.2$, $\tau_{D}=20$ and $V_{rev}=-80$ if the synapse originates from an O-LM cell, and $\tau_{R}=0.3$, $\tau_{D}=9$ and $V_{rev}=-80$, if the synapses originate from CA3 or DG interneurons.
